# Toxicoproteomics of APAP-exposed HepG2 cells identifies candidate translational biomarkers of liver injury and recovery

**DOI:** 10.3389/fphar.2026.1819323

**Published:** 2026-09-24

**Authors:** Ana S. Serras, Sérgio P. Camões, Rui Vitorino, Joana P. Miranda

**Affiliations:** 1 Research Institute for Medicines (iMed.ULisboa), Faculty of Pharmacy, Universidade de Lisboa, Lisbon, Portugal; 2 LAQV/REQUIMTE, Mass Spectrometry Center, Department of Chemistry, University of Aveiro, Aveiro, Portugal; 3 Department of Surgery and Physiology, Cardiovascular R&D Center, Faculty of Medicine, University of Porto, Oporto, Portugal; 4 Department of Medical Sciences, Institute of Biomedicine iBiMED, University of Aveiro, Aveiro, Portugal

**Keywords:** biomarker discovery platform, drug-induced liver injury, human *in vitro* model, paracetamol (APAP), toxicoproteomics, translational biomarkers

## Abstract

**Introduction:**

Drug‐induced liver injury (DILI) is a leading cause of acute liver failure (ALF) and a major obstacle in drug development, reflecting the lack of robust, mechanism‐based biomarkers. Human *in vitro* systems, coupled with unbiased omics, provide controlled, human‐relevant and mechanistically informative platforms that can accelerate biomarker discovery. Here, we explore quantitative toxicoproteomics of paracetamol (acetaminophen; APAP)‐exposed HepG2 cells as a platform for the identification of candidate translational biomarkers of APAP‐liver injury and recovery.

**Methods:**

HepG2 cells were exposed to the APAP IC_50_ concentration for 8 h (injury phase), followed by drug withdrawal and a 24 h recovery phase. Injury‐induced cell responses were evaluated through RT‐qPCR analysis and intracellular ROS quantification. Proteomic changes associated with injury and recovery were characterized by quantitative LC‐MS/MS and selected proteins were validated by immunoblotting.

**Results:**

LC‐MS/MS quantified 1,478 proteins and revealed increased protein secretion upon APAP exposure. Notably, comparison of the proteomic dataset against published proteomic profiles of a large cohort of ALF patients supported the potential clinical relevance of the obtained signature. Injury‐phase upregulated proteins, including KRT18 and HMGB1, recapitulated known clinical DILI biomarkers. Similarly, recovery‐phase was marked by clinical markers of liver regeneration, *e.g*., AFP, APOA1 and HP, enabling discrimination between injury and regeneration. HPD, S100A6 and TXNRD1 emerged as candidate diagnostic biomarkers, whilst SERPINA1 and complement components (C3, C5) showed prognostic potential.

**Conclusion:**

Collectively, this study demonstrated how toxicoproteomics applied to human *in vitro* hepatic models, validated against clinical evidence, provide a relevant platform for the discovery of novel translational biomarkers of liver injury and recovery.

## Graphical Abstract

**Figure:**
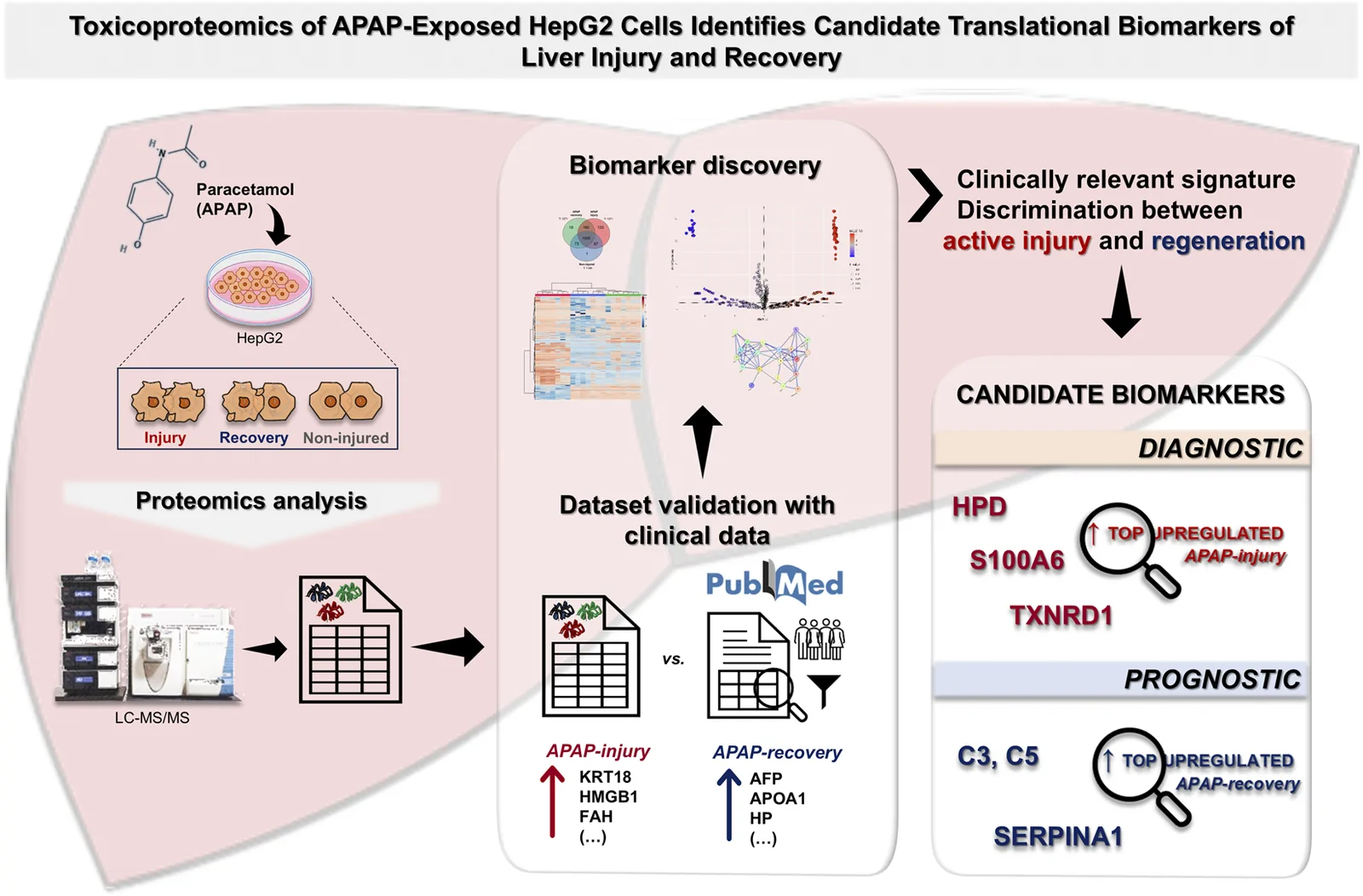

## 1 Introduction

Drug-induced liver injury (DILI) stands for a liver damage caused by drugs, over-the-counter medicines, xenobiotics or herbal products and constitutes one of the most common causes of acute liver failure (ALF) in Western countries (Teschke et al., 2017; Garcia-Cortes et al., 2020; Weiler et al., 2020; Fontana et al., 2023; Segovia-Zafra et al., 2024; Halegoua-DeMarzio and Navarro, 2025). Besides, it remains a major clinical and regulatory burden as it is the main culprit for drug failure in clinical trials, drug attrition or use restriction, partially due to the limited predictive capacity of preclinical *in vivo* models and lack of relevant biomarkers (Serras et al., 2021b; Segovia-Zafra et al., 2024). DILI leads to the elevation of serum liver enzymes, including aminotransferases (AST, ALT), alkaline phosphatase and total bilirubin, which currently form the basis for detection and monitoring. However, these biomarkers lack specificity for DILI and may be elevated in a wide range of other liver diseases. In addition, the heterogeneous clinical phenotypes of DILI often necessitate a diagnosis of exclusion, delaying recognition and increasing the risk of severe, potentially life-threatening outcomes (Andrade et al., 2019). Therefore, the search for novel molecular biomarkers for DILI diagnosis and prognosis, which would timely direct therapeutic interventions and prevent unnecessary liver transplantation, has gained increasing importance in clinical practice. Notably, biomarkers can serve a variety of purposes, namely, disease diagnosis (diagnostic biomarkers), evaluation of disease occurrence or progression (prognostic biomarkers), indication of treatment response (predictive biomarkers), amongst others (FDA-NIH Biomarker Working Group, 2016). Thus, the development of a new biomarker typically progresses from the phase of discovery, through confirmation in human cohorts and laboratory test validation, to eventual application in the clinical setup (Beger et al., 2015; Nguyen et al., 2022; Bader et al., 2023).

Paracetamol (APAP), one of the most widely used analgesic and antipyretic drugs, is safe and effective at therapeutic doses, but overdose causes severe liver injury and even death. In fact, APAP overdose is one of the most common causes of ALF, standing as a prototypical example of intrinsic DILI (Andrade et al., 2019). APAP toxicity*, in vivo,* occurs after the administration of high doses that lead to a shift from its main metabolization by phase II detoxifying enzymes to a metabolization by phase I enzymes (*e.g.,* CYP2E1), which leads to the formation of the reactive metabolite N-acetyl-p-benzoquinone imine (NAPQI). Glutathione effectively detoxifies NAPQI at therapeutic doses of APAP. However, in overdose conditions, the accumulation of NAPQI rapidly depletes glutathione pools, which leads to the covalent binding of the reactive metabolite to mitochondrial proteins, leading to increased reactive oxygen species (ROS) production. The activation of MAPK pathways with sustained JNK phosphorylation leads to hepatocyte necrosis and liver failure. Subsequently, necrotic hepatocytes release damage-associated molecular patterns (DAMPs) that trigger a sterile inflammation and further promote injury progression or initiation of liver regeneration (Woolbright and Jaeschke, 2017; Jaeschke et al., 2020; Yang et al., 2022). Nevertheless, current clinical tools fail to early diagnose and predict severe outcomes of APAP-associated DILI. Thus, in the past few years, human studies on the mechanisms of APAP toxicity have driven increased interest on new mechanistic-based candidate biomarkers. These include the high-mobility group box 1 (HMGB1), keratin-18 (KRT18) and liver-specific miR-122, which are released into the bloodstream after hepatocyte death or damage; glutamate dehydrogenase (GLDH), a mitochondrial enzyme primarily found in liver and released following mitochondrial toxicity; and APAP-protein adducts, formed after the generation of the reactive metabolite NAPQI, which allows to identify the APAP-oxidation metabolism. Most of these molecules have been detected before ALT elevation, showing not only diagnostic but also their improved prognostic value compared to ALT alone (McGill and Jaeschke, 2018; Church et al., 2019; Chiew et al., 2020; Umbaugh and Jaeschke, 2021). However, the development and incorporation of novel biomarker panels for DILI require rigorous and independent validation. The case of HMGB1 illustrates this need: although the European Medicines Agency (EMA) issued a Letter of Support for a panel of exploratory DILI biomarkers that included total and hyperacetylated HMGB1, this support was subsequently retracted following concerns regarding the reliability of data on hyperacetylated HMGB1. Consequently, HMGB1 isoforms cannot currently be regarded as clinically validated diagnostic biomarkers for DILI. Rather than replacing structured causality assessment, novel biomarkers should be evaluated as complementary tools that may support and corroborate DILI diagnoses established using the Roussel Uclaf Causality Assessment Method (RUCAM) (Teschke et al., 2017; 2019). Accordingly, none of these emerging DILI biomarkers are already established in clinical practice due to regulatory hurdles by lack of qualification processes, high variability or limited predictive power (Matheis et al., 2011; Church et al., 2019; Fu et al., 2020). This emphasizes the need for a shift from the measurement of a single marker into an integration of a panel of well-characterized biomarkers that would allow a timely and more effective identification and monitoring of disease. In this context, the use of alternative *in vitro* models combined with omics-based approaches are increasingly recognized as valuable tools to elucidate DILI mechanisms and to identify molecular signatures and translational biomarkers with potential predictive, diagnostic, or monitoring value (Prot et al., 2011; Van Summeren et al., 2011; Guo and van den Beucken, 2024).

*In vitro*, cell toxicity is also observed upon APAP exposure. However, several authors argue that, unlike the *in vivo* setting, APAP toxicity in traditional cultures of human, mouse, and rat hepatocytes is not dependent on NAPQI formation (Behrends et al., 2019; Livoti et al., 2025). Indeed, HepG2 cells, a human hepatic *in vitro* model widely used in liver toxicity studies, exhibit limited cytochrome P450 activity, including low CYP2E1 expression, which results in reduced formation of NAPQI following APAP overdose. Thus, APAP toxicity in HepG2 cells has been proposed to be driven not primarily by CYP2E1-mediated oxidative stress, but rather by metabolic and mitochondrial dysfunction contributing to cell death (Behrends et al., 2019). Despite these limitations, *in vitro* hepatocyte models remain high-throughput, human-relevant and mechanistically informative platforms. It allows a precise control of experimental conditions, overcoming the confounding factors associated to the overt systemic decompensation seen in DILI patients, thereby generating mechanistic and toxicity insights with translational relevance for biomarker discovery.

On the other hand, systems toxicology/biology -*omics* have emerged as powerful tools for the discovery of new molecular biomarkers. Proteins, as major constituents of body fluids, represent particularly valuable, stable and easily detectable targets (with methods currently used in the clinics) for the development of new translational biomarkers (Bader et al., 2023). Toxicoproteomics analyses of large cohorts of human biofluid samples already proposed proteins such as aldolase B (ALDOB), catalase (CAT), and polymeric immunoglobulin receptor (PIGR) as tentative biomarkers for discriminating APAP from non-APAP patients. Alpha-1-antitrypsin (SERPINA1), leucine-rich alpha-2-glycoprotein (LRG1) and lactate dehydrogenase (LDH) were further suggested as potential prognostic biomarkers, although still needing clinical validation (Vazquez et al., 2022; Remih et al., 2025).

Therefore, we describe a streamlined approach for the identification of candidate molecular biomarkers associated with liver injury and recovery using quantitative proteomics analysis of HepG2 cells exposed to APAP. In this study, HepG2 cells were exposed to APAP to induce hepatocyte injury mainly through metabolic and mitochondrial dysfunction, together with non-specific cytotoxic effects, thereby enabling the characterization of both injury- and post-injury-associated protein alterations. This approach aims to establish a simplified, controlled, and reproducible toxicoproteomics-based *in vitro* platform for biomarker discovery.

## 2 Methods

### 2.1 Reagents

All culture media and supplements, solvents (all analytical grade) and other chemicals were acquired from Sigma-Aldrich (Madrid, Spain) unless specified.

### 2.2 Cell culture

HepG2 cells were cultured in Minimum essential medium Eagle alpha modification (α-MEM) supplemented with 10% fetal bovine serum (FBS), 1 mM of sodium pyruvate and 1% of non-essential amino acids (NEAA) and incubated at 37 °C in a humidified atmosphere with 5% CO_2_. Cell viability was assessed through trypan blue exclusion method.

### 2.3 *In vitro* cell viability analysis

HepG2 were seeded at 6.0 × 10^4^ viable cells/cm^2^ in 96-well plates for 48 h. Afterwards, cells were exposed for 24 h to 5, 10, 20, 30, 50, 60, 70 and 90 mM of APAP (A11240, Alfa Aesar), directly dissolved in basal media without FBS, after which APAP cytotoxicity was evaluated using the MTS reduction assay (Promega®), according to manufacturer’s instructions. Three to five independent experiments in triplicates were performed. The absorbance was measured at 490 and 690 nm using microplate reader SPECTROstar Omega (BMG Labtech®). The estimated half-maximal inhibitory concentration (IC_50_) was calculated through a non-linear regression fit. Results were expressed as percentage relative to control, which was considered as 100% cell viability.

### 2.4 APAP injury establishment

HepG2 in standard monolayer cultures were seeded at 6.0 × 10^4^ viable cells/cm^2^ and maintained in α-MEM with 10% FBS, 1 mM of sodium pyruvate and 1% of NEAA for 48 h. After this, cells were incubated with 30 mM APAP (IC_50_) for 8 h in its basal medium without FBS (APAP-injury). To produce APAP-recovery, cells were washed two times with PBS after the 8 h APAP exposure and the medium was replaced by fresh basal medium without APAP and FBS in a final volume of 25 mL per 175 cm^2^ t-flask for other 24 h. HepG2 not exposed to APAP were collected in parallel at 32 h (8 h + 24 h) as control (Non-injured; Figure 1). APAP-injury, APAP-recovery and Non-injured were collected under sterile conditions and centrifuged at 300 *g* for 10 min at 25 °C, following 2700xg for 30 min at 4 °C. The collected conditioned media was concentrated using Amicon® Ultra-15 (Millipore®) 3 kDa cut-off, as per manufacture’s recommendations, and used for total protein quantification and proteomics analysis. Cells were collected for total protein quantification and gene expression analysis. Samples were stored at - 80 °C until further use.

**FIGURE 1:**
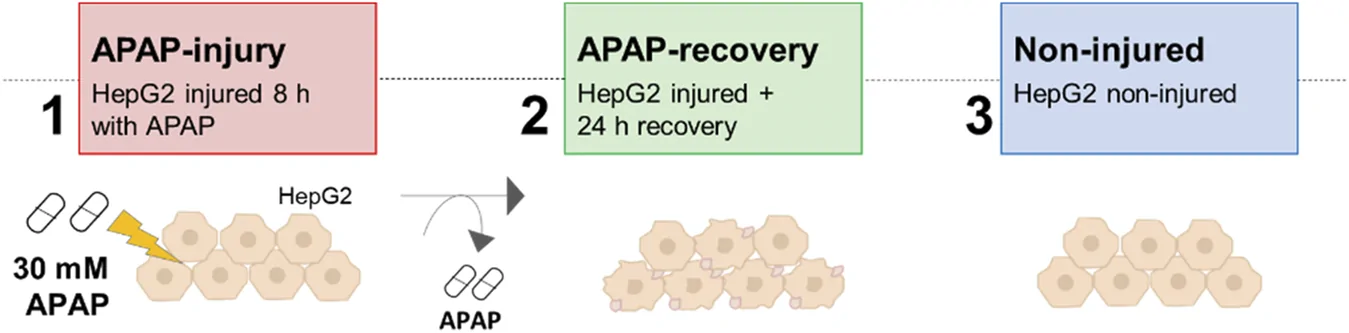
Schematic representation of the methodological approach for HepG2 injury with APAP and further recovery. APAP, paracetamol.

### 2.5 Total protein quantification

Total protein quantification of HepG2 cells and conditioned media was determined by the colorimetric assay based on the Bradford method, with protein dye reagent concentrate (500–0006, Bio-Rad®), according to manufacturer’s instructions for standard procedure for microtiter plates. Absorbance at 595 nm was measured using microplate reader on SPECTROstar® Omega.

### 2.6 Intracellular ROS levels

Intracellular ROS levels were determined using the dichloro-dihydro-fluorescein diacetate (DCF-DA) probe. Briefly, HepG2 were seeded in a black 96-well plate (Sarstedt) until reaching confluence, after which APAP-injury and APAP-recovery conditions were established. Upon exposure, for positive control, cells were treated with tert-butyl hydroperoxide (TBHP, 1 mM) for 1 h at 37 °C before probe incubation. Afterwards, cells were incubated with 10 μM of DCF-DA for 1 h at 37 °C. Fluorescence intensity was measured at excitation/emission = 460 nm/528 nm, using the Synergy HTX Multi-mode Microplate Reader (BioTek Instruments). Five independent experiments in quadruplicate were performed. Results were expressed as percentage relative to non-injured control, which was considered 100%.

### 2.7 Gene expression

Total RNA of 5.0 × 10^5^ HepG2 cells was isolated using TRIzol (Life Technologies) and extracted according to manufacturer’s instructions. RNA concentration was determined using LVis plate mode (SPECTROstar Omega) by measuring the absorbance at 260 nm. cDNA was synthesized from 1 μg of RNA using NZY First-Strand cDNA Synthesis Kit (NZYTech), according to manufacturer’s instructions. Quantitative real-time polymerase chain reaction (qRT-PCR) was performed using PowerUp SYBR Green Master Mix (Life Technologies) to a final reaction volume of 5 μL, using 1 μL of template cDNA and 0.333 μM of forward and reverse primers (Table 1). Reaction was performed on QuantStudio™ 7 Flex Real-Time PCR System (Applied Biosystems) as described by (Cipriano et al., 2017). Gene expression was quantified using the comparative Ct method (2^−ΔΔCt^) and normalized to the reference gene (*β-actin*).

**TABLE 1:** Primers used for qRT-PCR characterization of HepG2 gene expression.

Gene	Forward sequence (5′-3′)	Reverse sequence (5′-3′)
*β-actin*	CAT​GTA​CGT​TGC​TAT​CCA​GGC	CTC​CTT​AAT​GTC​ACG​CAC​GAT
*pdk4*	TCT​GAG​GCT​GAT​GAC​TGG​TG	GGA​GGA​AAC​AAG​GGT​TCA​CA
*glut1*	TCT​GGC​ATC​AAC​GCT​GTC​TTC	CGA​TAC​CGG​AGC​CAA​TGG​T
*fasn*	GTG​TCC​ACC​AGC​AAC​ATC​AG	GTT​CTC​CAG​CAA​GCC​ATC​TC

### 2.8 Western blot analysis

30 µg of total protein from conditioned media of HepG2 of each condition were resolved by SDS-PAGE in 12% polyacrylamide gels prepared as described by (Miranda et al., 2006). Gels were blotted onto PVDF membranes, which were incubated with primary antibody diluted in 5% bovine serum albumin overnight at 4 °C, namely, anti-TXNRD1 and anti-GDF15 diluted 1:200 (sc-20147 and sc-377195, respectively; Santa Cruz Biotechnology), anti-GSS diluted 1:1000 (ZRB2624; Sigma-Aldrich), and anti-SERPINA3 and anti-AZGP1 diluted 1:500 (MAB1295 and MAB4764, respectively; R&D Systems). Afterwards, membranes were washed and incubated with horseradish peroxidase-conjugated anti-mouse or anti-rabbit antibody diluted 1:20,000 (715–035-150 or 711–035-152, respectively; Jackson ImmunoResearch) for 1 h at room temperature. Protein loading control was performed with Ponceau S staining (Supplementary Figure S1). Immunoreactive bands were detected by enhanced chemiluminescence (Immobilon; Millipore) according to manufacturer’s instructions and images were recorded using an iBright™ CL750 Imaging System (Invitrogen).

### 2.9 LC-MS/MS analysis

7 replicates of each sample group were prepared for LC-MS/MS. LC-MS/MS Analysis was performed according to our previously described protocol (Serras et al., 2021a). Peptide mixtures were analyzed using a nanoLC system coupled to a high-resolution Orbitrap mass spectrometer (Thermo Fisher Scientific). Separation was achieved on a C18 column (75 μm × 25 cm, 2 µm particle size) with a linear gradient of 5%–35% ACN in 0.1% formic acid over 90 min at a flow rate of 300 nL min^-1^. The instrument operated in data-dependent acquisition mode with a top-20 method. Raw spectra were processed using MaxQuant (v2.2) against the *Homo sapiens* UniProt database. The mass spectrometry proteomics data have been deposited in the ProteomeXchange Consortium via the PRIDE (Perez-Riverol et al., 2019) partner repository with the dataset identifier PXD072667.

### 2.10 Proteomics data processing and bioinformatics

Data filtering was performed based on the identification of at least 2 unique peptides in each protein and the respective protein should be found in at least 5 replicates. All contaminants were removed. Data processing was performed through Log_2_ transformation of all intensities. List of significantly regulated proteins were calculated with PolyStest (Schwämmle et al., 2020) and statistical significance was considered with a threshold of false discovery rate (FDR)-adjusted *p* value <0.05. Proteins were considered unique when present in more than 4 replicates in the injury conditions whilst not identified in at least 6 replicates in the control condition.

Several web-available bioinformatic tools were used for identifying protein-protein interactions and their respective biological roles, namely, MetaboAnlayst 5.0 (Pang et al., 2022), GOrilla (Eden et al., 2009) and String-DB (Szklarczyk et al., 2019). List of liver enriched proteins was obtained from The Human Protein Atlas (Uhlén et al., 2015) and proteomics dataset from serum of acute hepatic failure patients was obtained from (Remih et al., 2025).

### 2.11 Statistical analysis

Results are presented as Average ± SD unless stated otherwise. IC_50_ was analyzed using GraphPad Prism. Statistical significance of the proteomics dataset was calculated with PolySTest (Schwämmle et al., 2020) and a threshold of FDR-adjusted *p* value <0.05 was considered statistically significant.

## 3 Results

### 3.1 Proteomic analysis of APAP-injured HepG2 cells exhibits biomarkers found in human plasma of DILI patients

To define the best concentration to produce APAP-injury conditions, concentration-response curves were performed using the MTS reduction assay for determination of IC_50_ values (Figure 2a). As expected, a 24 h exposure to APAP led to a concentration-dependent decrease of cell viability with an IC_50_ value of 30.12 ± 1.84 mM. Moreover, for the purpose of biomarkers identification, it is important to define i) a concentration able to cause cell stress and damage, but not severe cell death, and ii) a timepoint capturing early stress and injury-associated signaling without inducing extensive cell death. Therefore, cells were exposed to APAP at 30 mM for 8 h as previous *in vitro* studies already highlighted that hepatocytes exhibit the characteristic APAP toxicity mechanisms after 6 h of APAP exposure, after which cell death starts (Bajt, 2004; Yan et al., 2018). Importantly, exposure to APAP for 8 h did not lead to extensive cell death, with HepG2 exhibiting only a ∼14% decrease in total protein quantification (Figure 2b) and maintenance of cell morphology (Supplementary Figure S2). Moreover, 8 h of APAP exposure led to a decrease in reactive oxygen species (ROS) production (*p* < 0.0001; Figure 2c) and an upregulation of glycolysis- and glucose uptake-related genes, namely, *pdk4* (*p* < 0.0001) and *glut1* (*p* < 0.001), as well as a downregulation of lipogenesis-related genes, namely, *fasn* (*p* < 0.05; Figure 2d). Likewise, concordant with the metabolic stress toxicity mechanism previously proposed for APAP toxicity in HepG2 cells (Behrends et al., 2019), upon APAP exposure, the proteomics analysis also showed an increased secretion of proteins related to glycolysis, such as GAPDH, ALDOA and GOT1, antioxidant defense, such as TXNRD1, GSR and GSS, and stress-inducible chaperones, such as HSPA5 and HSP90AA1 (Figure 2e).

**FIGURE 2:**
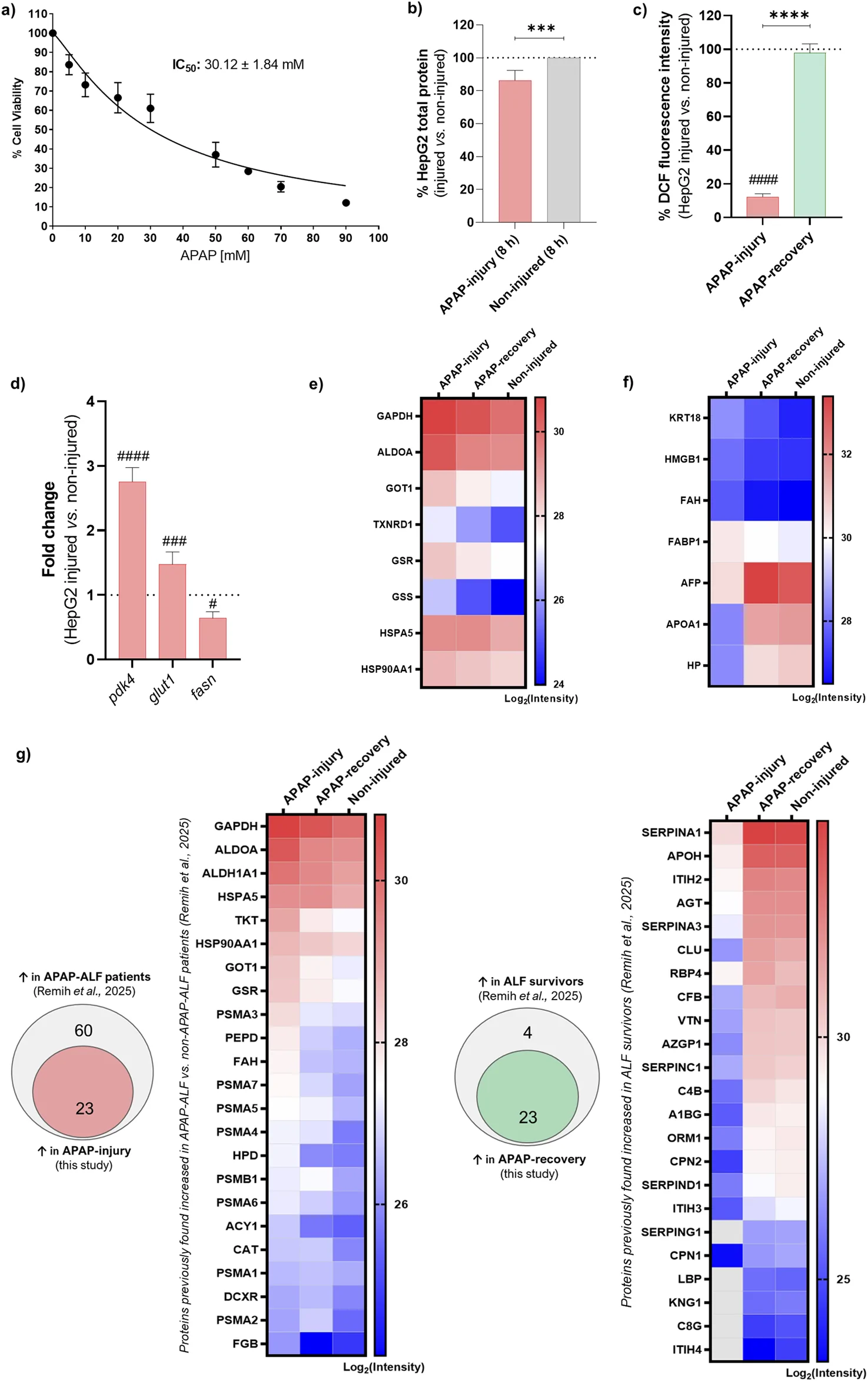
Exposure of HepG2 to APAP led to a decrease in cell viability without extensive cell death as well as a decrease in reactive oxygen species (ROS) production, an upregulation of glycolysis- and glucose uptake-related genes and an increased secretion of proteins typically found in DILI patients’ plasma. **(a)** Cell viability of HepG2 cells exposed to different concentrations of APAP (0–90 mM) for 24 h, as assessed by the MTS reduction assay (values are expressed relative to non-treated cells; n = 3–5). **(b)** Total protein quantification of HepG2 cells exposed to 30 mM of APAP for 8 h (APAP-injury), as assessed by Bradford protein assay (values are expressed relative to non-treated cells; n = 5). **(c)** ROS levels of HepG2 after 8 h of exposure to APAP (red) and after 24 h of APAP withdrawal (green) (values are expressed relative to non-treated cells; n = 5), using the DCF-DA fluorescent probe. **(d)** Gene expression levels of *pdk4*, *glut1*, and *fasn* (values are expressed relative to non-treated cells and represented as Average ± SEM; n = 3–4). Data is normalized to the reference gene *b-actin*. **(e)** Heatmap displaying protein expression associated with glycolytic metabolism, antioxidant response and stress-inducible chaperones. **(f)** Heatmap showing proteins specifically associated to DILI. **(g)** Euler diagrams and heatmaps showing proteins overlapping between APAP-injury and APAP-induced ALF patients from (Remih et al., 2025) (left), and between APAP-recovery and ALF patient survivors from (Remih et al., 2025) (right). Statistical significance as ***p < 0.001 and ****p < 0.0001; and ^#^ *p* < 0.05; ^###^ *p* < 0.001 and ^####^ *p* < 0.0001, compared to non-injured control. APAP-injury, HepG2 cell line exposed to APAP for 8 h; APAP-recovery, HepG2 cell line exposed to APAP for 8 h, followed by APAP withdrawal for 24 h; non-inured, HepG2 cell line non-exposed to APAP; ALF, acute hepatic failure; APAP, paracetamol; DILI, drug-induced liver injury; IC_50_, half-maximal inhibitory concentration; ROS, reactive oxygen species.

To better resemble clinical DILI progression, two groups were defined, namely, the *APAP-injury*, representing 8 h after APAP exposure, and *APAP-recovery*, which corresponds to 24 h after APAP withdrawal. Non-injured HepG2 (group 3) were used as control (Figure 1, methods section).

Importantly, to evaluate whether the proteomic profile of APAP-injured HepG2 cells reflected a DILI context, we first screened for proteins previously reported in samples from DILI patients. Proteins such as keratin-18 (KRT18), high-mobility group box-1 (HMGB1), fumarylacetoacetate hydrolase (FAH) and fatty acid-binding protein (FABP1) have been reported to be elevated in the serum or plasma of DILI patients (Antoine et al., 2013; Dear et al., 2018; Church et al., 2019; Ravindra et al., 2023). In contrast, alpha-fetoprotein (AFP), apolipoprotein A1 (APOA1) and haptoglobin (HP) are known to be increased in patients that recovered from APAP-induced liver injury (Peta et al., 2017; Church et al., 2019). Notably, this same trend was observed in our proteomics dataset, with the former proteins showing a higher quantity in the APAP-injury group, which decreases towards the non-injured condition. Likewise, AFP, APOA1 and HP were decreased in the APAP-injury group and augmented during recovery, as depicted in Figure 2f and Supplementary Table S1. Besides, when compared to the large clinical proteomics study of Remih et al., 2025), several proteins that were upregulated in our APAP-injury group were also found significantly upregulated specifically in the APAP-DILI patients (vs. other ALF patients), such as ALDOA, glutathione reductase (GSR), CAT, amongst others (Figure 2g left; Supplementary Table S1). Most importantly, almost all proteins (23 out of 27) previously found significantly downregulated in ALF patients that did not survive or had to undergo transplantation (vs. 21-day transplant-free survivors), were found to be upregulated in our APAP-recovery condition, *e.g.,* SERPINA1, alpha-antichymotrypsin (SERPINA3), apolipoprotein H (APOH) or zinc-alpha-2-glycoprotein (AZGP1; Figure 2g right; Supplementary Table S1).

### 3.2 APAP withdrawal drives proteomic remodeling from injury to recovery and reflects intermediate recovery states

The proteomics analysis displayed a variety of 1478 different proteins, in which 1005 were shared between all groups and 160 shared only within injury conditions. Moreover, injury with APAP led to a higher secretion of unique proteins, which decreased during recovery (19 proteins) until the non-injured state (1 protein; Figure 3a). Likewise, when compared with the basal state, APAP exposure stimulated a higher expression of shared proteins, with the APAP-injury group exhibiting 614 significantly upregulated (and 155 significantly dowregulated) followed by APAP-recovery with 211 significantly upregulated (and 56 significantly dowregulated; Figure 3b).

**FIGURE 3:**
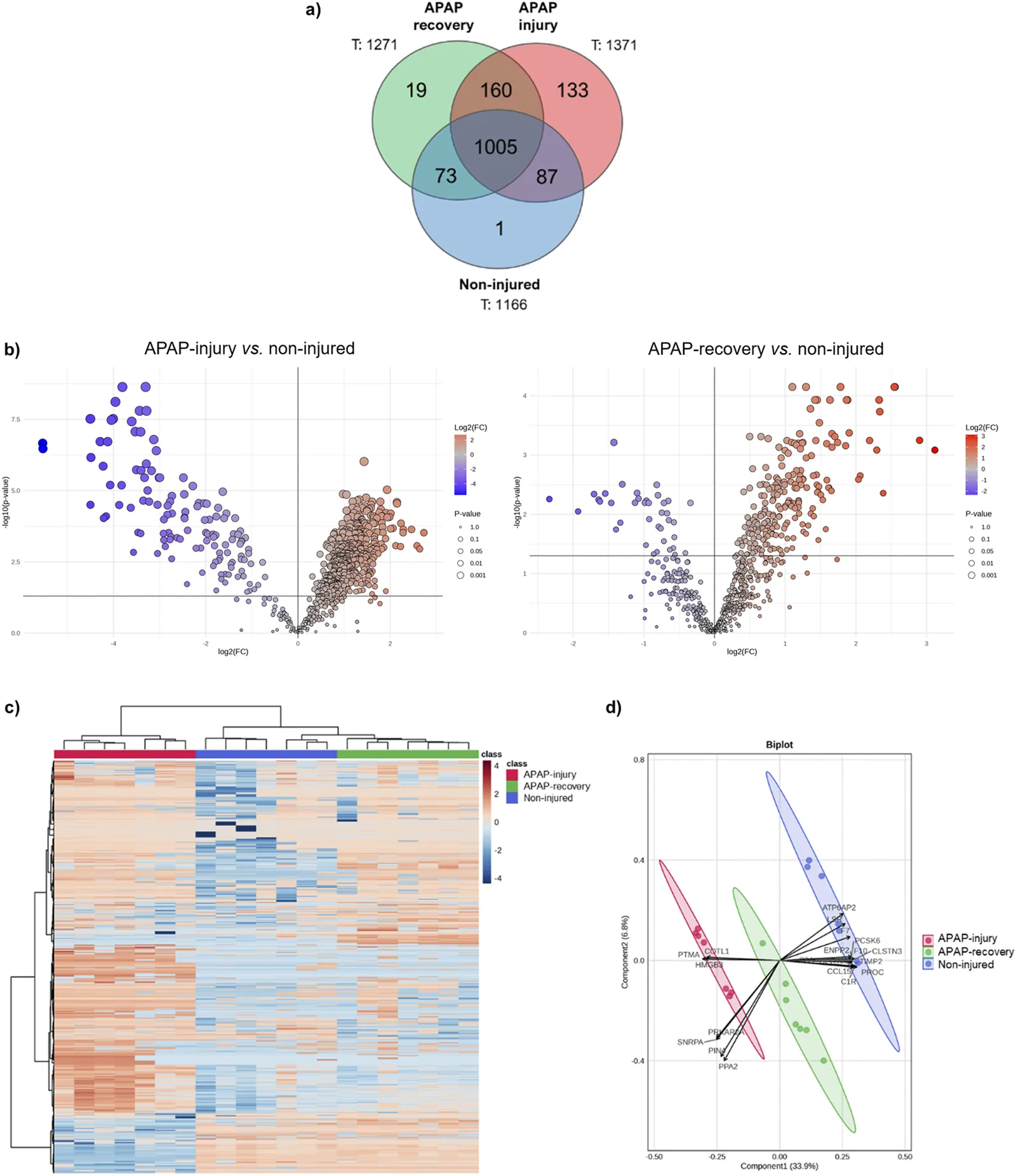
Proteomic profile analysis of APAP-injury and APAP-recovery. **(a)** Venn diagram, **(b)** volcano plot depicting up/downregulated proteins within groups, **(c)** hierarchical clustering heatmap, and **(d)** PLS-DA biplot of proteomic dataset. Different samples were discriminated with high predictive power (Q^2^ > 0.8); n = 7. APAP-injury, HepG2 cell line exposed to APAP for 8 h; APAP-recovery, HepG2 cell line exposed to APAP for 8 h, followed by APAP withdrawal for 24 h; non-injured, HepG2 cell line non-exposed to APAP; APAP, paracetamol; FC, fold-change; FDR, false discovery rate; T, total.

Besides, all sample classes grouped differently, with APAP-injury showing the highest difference with the basal state, but a similar profile to APAP-recovery, which, in turn, showed a higher similarity to the non-injured control (Figure 3c). This was further confirmed by the PLS-DA model, which discriminated APAP-injury from APAP-recovery and non-injured control with high predictive power (Q^2^ > 0.8; Figure 3d).

### 3.3 Exposure of HepG2 to APAP enables the identification of top unique and regulated proteins for biomarker selection

We next assessed all statistically significant up/downregulated and unique proteins between groups. Cells exposure to APAP led to a higher secretion of proteins related to RNA metabolism and translation, whereas, in the regenerative state, an upregulation of proteins related to the immune system process and extracellular structure organization were enriched when compared both to the APAP-injury or to the basal state (Figure 4a; Supplementary Figures S3, S4a,b). Similarly, KEGG pathways of complement and coagulation cascades as well as lysosome were enriched in the APAP-recovery group regardless of which group is compared (Figure 4b; Supplementary Figures S4c,d).

**FIGURE 4:**
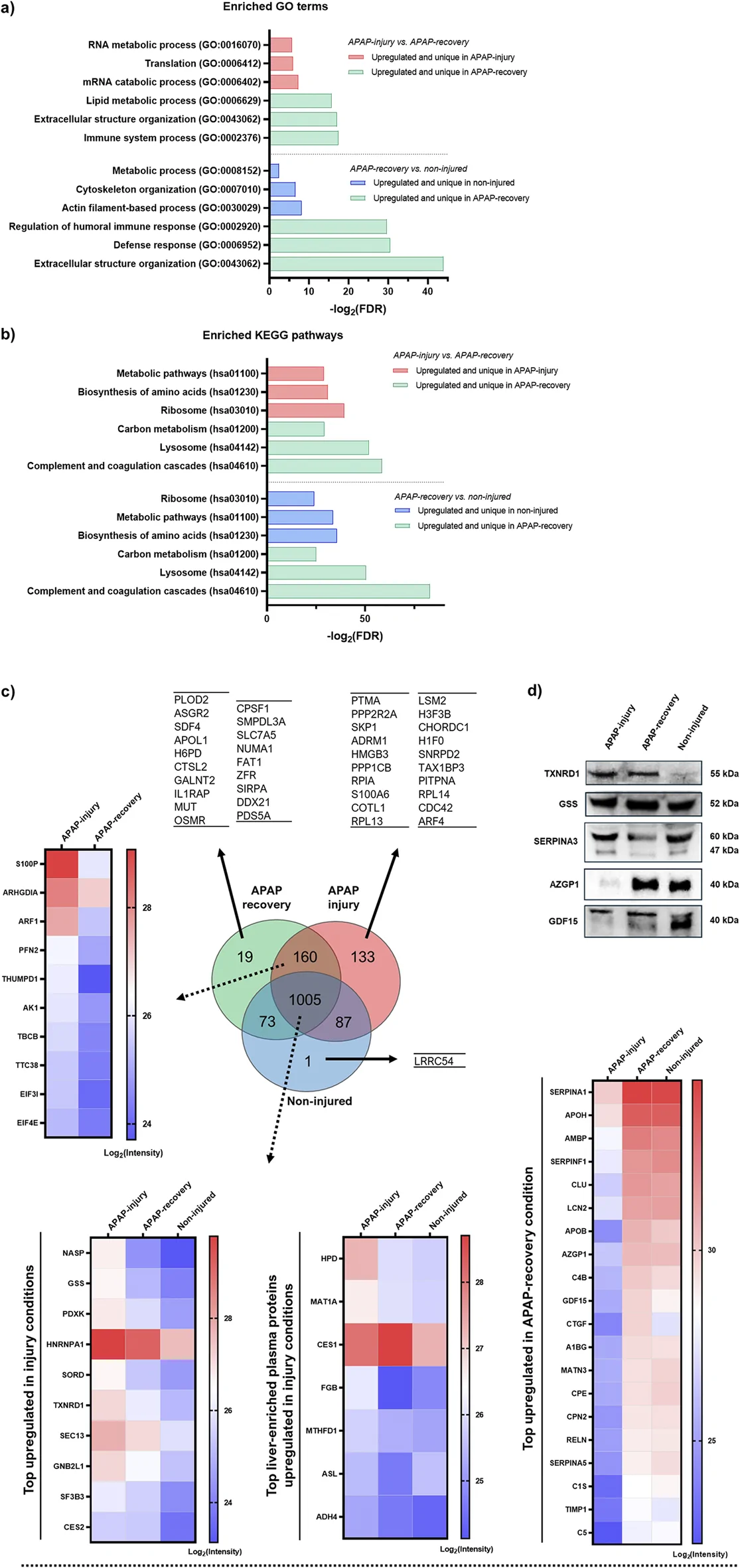
Exploring top unique and regulated proteins allows to identify new potential biomarkers. Top enriched **(a)** biological process GO terms and **(b)** KEGG pathways from APAP-injury compared to APAP-recovery and APAP-recovery compared with non-injured. **(c)** Venn diagram of proteomics dataset identifying top unique and significantly regulated proteins (represented in heatmap); **(d)** Western blot of selected proteins, shared between all groups, for validation of the proteomics dataset. APAP-injury, HepG2 cell line exposed to APAP for 8 h; APAP-recovery, HepG2 cell line exposed to APAP for 8 h, followed by APAP withdrawal for 24 h; non-injured, HepG2 cell line non-exposed to APAP; APAP, paracetamol; FDR, false discovery rate; GO, gene ontology.

For biomarker selection, top unique and significantly regulated proteins were screened (Figure 4c; Supplementary Table S2). Top intensity proteins, both unique and upregulated in injury conditions, may serve as diagnostic biomarkers, such as S100A6, unique to the APAP-injury, S100P, upregulated in APAP-injury compared to APAP-recovery (*p* < 0.0001) and absent in non-injured cells, and thioredoxin reductase 1 (TXNRD1), upregulated in both injury conditions compared to the basal state. Additionally, a deeper comparison of only liver-enriched plasma proteins constant in *The Human Protein Atlas* (Uhlén et al., 2015) enabled the identification of hydroxyphenylpyruvate dioxygenase (HPD), shown to be upregulated in the APAP-injury condition (*p* < 0.001). On the other hand, downregulated proteins in the APAP-injury condition and upregulated in APAP-recovery, such as SERPINA1 and complement proteins (*e.g.,* C5, C3; *p* < 0.0001) may represent prognostic biomarkers. The validation of the proteomic dataset was performed through qualitative Western blot analysis of selected proteins, namely, TXNRD1, glutathione synthetase (GSS), SERPINA3, AZGP1 and growth differentiation factor 15 (GDF15), which further confirmed its presence in all sample groups (Figure 4d; Supplementary Figure S5).

## 4 Discussion

Biomarkers have become a cornerstone of modern medicine, enabling accurate diagnosis and prognosis, stratification of risk, and more efficient disease management. They provide a faster, more precise and effective tool to identify disease, guide targeted therapies, and optimize healthcare resources. In the context of DILI, such biomarkers constitute key resources for predicting hepatotoxicity both during drug development and in the clinical setting following drug commercialization. Being APAP a prototypical drug that causes predictable DILI, the data generated from its toxicity studies provides a valuable reference framework for the development and validation of biomarkers applicable to other drug-induced liver injury agents. Yet, most non-clinical studies reported in literature focus on different dosing regimens and exposure time points, typically comparing drug-exposed with non-exposed cells or animals (Van Summeren et al., 2011; Gao et al., 2017; Driessen et al., 2022). While such approaches are essential, they primarily capture molecular events at disease onset. To our knowledge, this study is the first *in vitro* investigation to systematically integrate and compare against human biofluid samples all three stages–drug exposure (injury), withdrawal (recovery) and healthy controls–thereby enabling a more comprehensive assessment of disease progression and the identification of biomarkers with not only diagnostic but also prognostic value.

In this work, we firstly set up the *in vitro* conditions to model APAP-induced hepatic injury using HepG2 cells. Cell viability assays demonstrated a concentration-dependent cytotoxic effect of APAP displaying an IC_50_ of 30.12 ± 1.84 mM, consistent with previously reported values for this cell type and comparable culture conditions (Wang et al., 2002; Ramaiahgari et al., 2014; Deng et al., 2019). However, appropriate model development also requires consideration of human-relevant exposure levels (maximum plasma concentration; C_max_). Reported C_max_ of APAP ranges from 165 µM (therapeutic), to 463 µM (toxic) and 992 µM (comatose-lethal) (Regenthal et al., 1999). Given that *in vitro* systems typically require APAP concentrations 20- to 100-fold higher than human C_max_ values to account for differences in metabolism, exposure, and cellular context (Serras et al., 2021b), the IC_50_ obtained in our study falls within a physiological range encompassing both therapeutic and toxic exposures. Importantly, hepatocellular responses to APAP are influenced not only by concentration but also by exposure duration. *In vitro* models often exhibit an earlier toxicity response than that observed *in vivo*, not only due to the lack of the same multicellular and organ complexity, but also because cells are typically exposed to constant concentrations of the compound without the clearance that occurs *in vivo* (Serras et al., 2021b; Dichamp et al., 2023). Previous *in vitro* studies have shown that within the first 6 h of APAP exposure, hepatocytes already exhibit glutathione depletion, oxidative stress, mitochondrial dysfunction, protein adduct formation, and activation of c-Jun N-terminal kinase (*p*-JNK), whereas longer exposures are associated with overt cell death (Bajt, 2004; Yan et al., 2018). Thus, in this study, an 8 h exposure period was selected to allow interrogation of early injury mechanisms while preserving cellular integrity for subsequent recovery analyses.

Exposure of HepG2 to APAP for 8 h led to a decrease in ROS production as well as an upregulation of genes related to the inhibition of pyruvate entry into the tricarboxylic acid (TCA) cycle (*pdk4*) and increased glucose uptake (*glut1*), and downregulation of genes related to *de novo* lipogenesis (*fasn*). This pattern was further supported by an increase in the secretion of several proteins typically involved in metabolic reprogramming, including those that support enhanced glycolysis (GAPDH, ALDOA and GOT1), compensatory NADPH production (GOT1), adaptive redox responses (TXNRD1, GSR and GSS) and cell survival mechanisms (HSPA5 and HSP90AA1). It has already been suggested that APAP toxicity in HepG2 can be independent of oxidative stress but rather due to a metabolic stress response associated to a growth inhibition without extensive cell death (Behrends et al., 2019). Thus, the observed decrease in ROS after APAP exposure may instead reflect an adaptive or compensatory response to cell injury. Moreover, our results are concordant with the mechanism described by Behrends et al., 2019, in which APAP toxicity in HepG2 can be mainly marked by an increase in glycolysis and lactate production, decoupling of glycolysis from the TCA cycle, NADPH depletion and reduced *de novo* fatty acid synthesis (Behrends et al., 2019).

Building on evidence from patient-derived samples, several biomarkers have emerged as early indicators of hepatocellular injury. Consistent with clinical observations, our dataset showed an enrichment of relevant proteins detectable in plasma or serum of DILI patients. Specifically, the APAP-injury condition was marked by increased abundance of typical injury-associated proteins such as KRT18 and HMGB1, FAH and FABP1 (Antoine et al., 2013; Dear et al., 2018; Church et al., 2019; Ravindra et al., 2023), alongside with reduced levels of AFP, APOA1 and HP (Peta et al., 2017; Church et al., 2019). Both KRT18 and HMGB1, although lacking liver specificity, can be measured in blood of APAP-liver injury patients and correspond to widely studied early-stage injury markers. KRT18 is an indicator of the cell death mode (necrosis or apoptosis) and has been investigated as potential diagnostic and prognostic biomarker of APAP-induced liver injury (Antoine et al., 2013; Church et al., 2019). The HMGB1 protein is a DAMP typically found in the cell nucleus that is secreted after necrosis, being involved in APAP-induced sterile inflammation (Yang et al., 2022).

Interestingly, Remih et al. (2025) presented a large collaborative working group-based, multicenter, clinical proteomics study that collected and analysed, by untargeted proteomics, serum samples from 319 ALF patients (of which 50% constituted APAP-DILI patients). This study enabled the identification of several proteins associated with APAP patients (vs. other ALF pathologies) or with 21-day transplant-free survivors. Importantly, when compared to this clinical proteomics dataset, several proteins upregulated in our APAP-injury group, were also found upregulated specifically in APAP-induced liver injury patients, such as ALDOA, GSR, CAT, amongst others. This reciprocal regulation reflects a shift toward an active injury phenotype and supports the ability of our *in vitro* model to capture clinically relevant molecular signatures associated with both tissue damage and impaired regenerative capacity. Besides, herein, APAP exposure led to a higher secretion of proteins that were subsequently decreased during HepG2 recovery phase. During the active injury state (APAP-injury), there was a significant increase in proteins related to of RNA metabolism and translation pathways. This finding is consistent with evidence that APAP promotes a reprogramming of the cells’ transcriptome, including RNA modifications and translational responses that can either be deleterious or protective within the context of APAP toxicity (Shortt et al., 2019; Evke et al., 2022).

Importantly, when proposing novel translational biomarkers, several key criteria must be considered to ensure clinical applicability and relevance. First, candidate biomarkers should be detectable through minimally invasive sampling, such as in plasma or serum, to facilitate routine clinical use. Second, biomarkers should be mechanistically linked to liver injury pathways to ensure biological plausibility and disease specificity. Additional considerations include robustness across experimental systems, dynamic responsiveness to injury and recovery, and consistency with observations in patient samples (Beger et al., 2015; Remih et al., 2025). Together, these criteria provide a rational framework for prioritizing biomarker candidates with both diagnostic and prognostic potential in drug-induced liver injury. Thus, by analyzing the top unique and upregulated proteins in injury conditions, 3 potentially interesting circulating proteins arise as potential diagnostic biomarkers, namely S100A6, S100P and TXNRD1. The extracellular proteins of the S100 family act as DAMPs (Gonzalez et al., 2020) which are released after hepatocyte death and stimulate the inflammatory response (Yang et al., 2022). Specifically, S100A6 has been shown to be increased in mice models of APAP overdose, of which the *in vitro* mechanism was attributed to the activation of Kupffer cells through *p*-P38 and *p*-JNK signaling (Tong et al., 2023). TXNRD1 is one of the major redox modulator proteins and has been proposed as a physiological inhibitor of ASK1 (Saitoh, 1998), the MAPK kinase that activates JNK before APAP-induced hepatocyte necrosis. Besides, it has been shown to be upregulated in rats treated with APAP (Minami et al., 2005; Zidek et al., 2007). Nevertheless, although these mechanistical insights provide evidence that S100 and TNXRD1 proteins could constitute potential biomarkers, there is still, up to date, no human clinical studies supporting this. Furthermore, liver-specific proteins that were elevated in APAP-injury could aid in the identification of DILI. Within top upregulated liver-specific plasma proteins, HPD was highly expressed in the APAP-injury group. It is involved in tyrosine catabolism and has already been found augmented in serum and plasma of mice and human patients of APAP liver injury (Hu et al., 2014; Beger et al., 2015), being also significantly increased in APAP-induced ALF patients compared with other types of ALF (Remih et al., 2025), heightening the possibility of its use as specific biomarker for APAP overdose. Nevertheless, contrary to the former proteins, there are still no mechanistic studies that can explain HPD involvement in APAP-liver injury.

Following APAP withdrawal, cells displayed a distinct injury-response profile characterized by increased secretion of proteins related to immune system processes. This response may be linked to the release of damage-associated molecules from necrotic hepatocytes, thereby triggering sterile inflammation, a critical determinant of both injury progression or initiation of liver regeneration (Yang et al., 2022). Moreover, pathways such as extracellular structure organization were also enriched on the APAP-recovery phase, further supporting maintenance of the response to APAP injury. Processes such as extracellular matrix remodeling, for example, are an integrant part of the liver regeneration process (Shi and Line, 2020), underscoring the biological relevance of the proteomic signatures observed during recovery. Importantly, restoration of AFP, APOA1, and HP levels during the APAP-recovery phase parallels patterns reported in patients with good prognosis, reinforcing the prognostic relevance of these proteins. AFP is widely regarded as a late-injury marker, with its post-necrotic elevation closely linked to hepatocyte proliferation and liver regeneration. Indeed, increased AFP levels have been observed in APAP liver injury patients with favorable clinical outcome, supporting its association with regenerative processes rather than ongoing hepatocellular damage (Schmidt and Dalhoff, 2005). Similarly, higher APOA1 and HP–both acute-phase proteins involved in lipid transport, immune modulation, and tissue repair - have been reported in DILI patients, including those with APAP overdose, who subsequently recovered (Peta et al., 2017). Besides, the fact that most of the proteins found increased in serum from surviving ALF patients (Remih et al., 2025) were also found upregulated in our APAP-recovery group, *e.g.,* SERPINA3, APOH and AZGP1, further supports its interpretation as a regenerative state. Accordingly, analysis of proteins downregulated during the APAP-induced injury phase, but upregulated during recovery, together with KEGG pathway enrichment of the recovery state, identified SERPINA1 and complement components (*e.g.,* C3 and C5) as potential prognostic biomarkers, as these are suppressed during early injury and subsequently restored during hepatic regeneration. Both are acute-phase proteins regulated by the IL-6/STAT3 pathway (Bode et al., 2012), which is involved in hepatocyte regeneration. SERPINA1 is a major protease inhibitor that have already shown anti-inflammatory and hepatoprotective effects on mice models of APAP overdose (Jedicke et al., 2014; Shabat et al., 2018). Notably, SERPINA1 showed a strong discriminative power between ALF survivors and non-survivors/transplanted in the study of Remih et al. (2025). Furthermore, the levels of complement proteins in plasma are a consequence of a balance between synthesis and consumption. In the context of liver failure, most complement proteins are reduced, whereas, during hepatocyte regeneration, the activation of complement proteins such as C3 and C5 are needed for liver repair (Thorgersen et al., 2019). In specific, C3 has been found to be depleted at early APAP injury but its levels were replenished over time in APAP overdose patients that survived (Woolbright et al., 2022). Importantly, the ability to capture both an active injury state (APAP-injury) and a subsequent regenerative phase (APAP-recovery) enabled the identification of biomarkers that reflect not only hepatocellular damage, but also recovery dynamics. Such temporal resolution is critical for improving clinical risk stratification and underscores the value of quantitative toxicoproteomics for identifying translational biomarkers that reflect disease progression in DILI.

## 5 Conclusion

Our results provide a comprehensive understanding of how quantitative proteomics data analysis of human *in vitro* models endorsed against human DILI samples can disclose new potential molecular biomarkers involved in liver injury and recovery. By modeling distinct stages of APAP-induced hepatotoxicity in HepG2 cells, we were able to corroborate the presence of several clinically relevant proteins within the context of APAP liver injury, including KRT18, HMGB1, AFP, APOA1 and HP, and propose additional candidate diagnostic biomarkers such as HPD, S100A6 and TXNRD1, and candidate prognostic biomarkers such as SERPINA1 and complement proteins, for further validation. Importantly, clinical validation of these candidate biomarkers must be performed in large and well-characterized cohorts of patients with rigorous causality assessment using diagnostic algorithms such as RUCAM. Despite the limitations of this *in vitro* model, this study provides a discovery-level, comprehensive platform to guide biomarker discovery *in vitro*, from model establishment through clinical validation and biomarker identification, supporting its translation to other advanced hepatic *in vitro* models and potentiating the discovery of novel protein biomarkers of both liver injury and recovery. When extending this workflow to study idiosyncratic DILI, additional factors such as genetic susceptibility (*e.g.,* specific HLA genotypes), immune response and drug metabolism activity (*e.g.,* CYP-mediated bioactivation), should be considered to better capture DILI complexity. Combined with clinical validations, this strategy can offer a relatively fast and robust approach for the identification of biomarker proteins across a wide range of diseases.

## Data Availability

The datasets presented in this study can be found in online repositories. The names of the repository/repositories and accession number(s) can be found below: http://www.proteomexchange.org/, PXD072667.

## Glossary

AFP: Alpha-fetoprotein
ALDOB: Aldolase B
ALF: Acute hepatic failure
APAP: Acetaminophen/paracetamol
APAP-injury: Conditioned media of HepG2 exposed to paracetamol for 8 h
APAP-recovery: Conditioned media of HepG2 24 h after paracetamol withdrawal
APOA1: Apolipoprotein A1
APOH: Apolipoprotein H
AZGP1: Zinc-alpha-2-glycoprotein
CAT: Catalase
**C**_**max**_: Maximum plasma concentration
DAMP: Damage-associated molecular patterns
DILI: Drug-induced liver injury
EMA: European Medicines Agency
FABP1: Fatty acid-binding protein
FAH: Fumarylacetoacetate hydrolase
FBS: Fetal bovine serum
FDR: False discovery rate
GDF15: Growth differentiation factor 15
GLDH: Glutamate dehydrogenase
GSR: Glutathione reductase
GSS: Glutathione synthetase
HMGB1: High-mobility group box 1
HP: Haptoglobin
HPD: Hydroxyphenylpyruvate dioxygenase
**IC**_**50**_: Half-maximal inhibitory concentration
KRT18: Keratin-18
LDH: Lactate dehydrogenase
LRG1: Leucine-rich alpha-2-glycoprotein
NAPQI: N-acetyl-p-benzoquinone imine
NEAA: Non-essential amino acids
PIGR: Polymeric immunoglobulin receptor
p-JNK: c-Jun N-terminal kinase
RUCAM: Roussel Uclaf Causality Assessment Method
SERPINA1: Alpha-1-antitrypsin
SERPINA3: Alpha-1-antichymotrypsin
TXNRD1: Thioredoxin reductase 1
α-MEM: Minimum essential medium Eagle alpha modification.
