# Supplementary material for: Toxicoproteomics of APAP-exposed HepG2 cells identifies candidate translational biomarkers of liver injury and recovery
Source: Front Pharmacol. 2026 Sep 24;17:1819323. doi: 10.3389/fphar.2026.1819323 (PMC13645762; doi:10.3389/fphar.2026.1819323)
Supplement: Supplementary file 1 [file Supplementaryfile1.docx]

Supplementary Material

# 1 Supplementary Figures and Tables

## 1.1 Supplementary Figures

**Supplementary Fig. 1.** Ponceau S-stained PVDF membranes. (**a**) Membrane used for TXNRD1 and AZGP1 detection; (**b**) membrane used for GSS, GDF15 and SERPINA3 detection. Lanes 1 and 5: molecular weight ladder; lane 2: HepG2 APAP-injury; lane 3: HepG2 APAP-recovery; lane 4: HepG2 non-injured.

**Supplementary Fig. 2.** Representative images of HepG2 cells (**a**) exposed and (**b**) not exposed to APAP for 8 h; (**a’**, **b’**) followed by a 24 h recovery period after APAP withdrawal/medium change. Scale bar = 200 µm.

**Supplementary Fig. 3.** Proteins upregulated in the APAP-injury condition related to the (**a**) enriched GO biological process terms RNA metabolic process (GO:0016070) and translation (GO:0006412); (**b**) as well as to the KEGG metabolic pathways (hsa01100). GO, Gene Ontology; KEGG, Kyoto Encyclopedia of Genes and Genomes.

**Supplementary Fig. 4.** Proteins upregulated in the APAP-recovery condition related to the enriched GO biological process terms of the (**a**) immune system process (GO:0002376) and (**b**) extracellular structure organization (GO:0043062); as well as to the KEGG pathways of (**c**) complement and coagulation cascades (hsa04610), and (**d**) lysosome (hsa04142). GO, Gene Ontology; KEGG, Kyoto Encyclopedia of Genes and Genomes.

**Supplementary Fig. 5.** Relative protein levels of TXNRD1, GSS, SERPINA3, AZGP1 and GDF15. (**a**) Densitometric analysis of Western blot bands, normalized to total protein staining (Ponceau S), compared to the non-injured condition (grid line equals to 1); (**b**) Fold-change in protein abundance of LC-MS/MS data analysis, compared to the non-injured condition.

## 1.2 Supplementary Tables

**Supplementary Table 1.** Fold-change (FC) and false discovery rate (FDR) of proteins found on plasma or serum samples of acute hepatic failure patients, including drug-induced liver injury. Protein list was retrieved from^a,b,c,d,e,f^. Statistical significance was calculated using PolySTest^g^. NA, non-applicable.

| **Gene name** | **Protein name** | **APAP-injury *vs.* APAP-recovery** | | **APAP-injury *vs.* non-injured** | | **APAP-recovery *vs.* non-injured** | |
| --- | --- | --- | --- | --- | --- | --- | --- |
|  |  | **Log_2_(FC)** | **FDR** | **Log_2_(FC)** | **FDR** | **Log_2_(FC)** | **FDR** |
| **KRT18** | Keratin, type I cytoskeletal 18 | 0.8067 | 1.67E-02 | 1.5537 | 5.93E-05 | 0.7470 | 1.26E-01 |
| **HMGB1** | High mobility group protein B1 | 0.7126 | 1.27E-03 | 0.8826 | 8.51E-04 | 0.1699 | 8.45E-01 |
| **FAH** | Fumarylacetoacetase | 0.9875 | 1.72E-04 | 1.1781 | 1.16E-05 | 0.1906 | 8.44E-01 |
| **FABP1** | Fatty acid-binding protein | 0.3796 | 2.05E-01 | 0.6225 | 5.60E-02 | 0.2428 | 8.99E-01 |
| **AFP** | Alpha-fetoprotein | -2.8177 | 1.44E-07 | -2.3667 | 2.99E-06 | 0.4509 | 5.59E-01 |
| **APOA1** | Apolipoprotein A-I | -3.2741 | 4.20E-12 | -3.4115 | 1.26E-12 | -0.1374 | 2.44E-01 |
| **HP** | Haptoglobin | -2.2255 | 1.71E-06 | -2.5052 | 2.86E-07 | -0.2797 | 5.65E-01 |
| **GAPDH** | Glyceraldehyde-3-phosphate dehydrogenase | 0.3561 | 1.38E-01 | 0.8672 | 1.46E-03 | 0.5111 | 9.60E-02 |
| **ALDOA** | Fructose-bisphosphate aldolase A | 0.8369 | 1.45E-05 | 0.9836 | 2.07E-06 | 0.1467 | 1.22E-01 |
| **ALDH1A1** | Retinal dehydrogenase 1 | 0.3670 | 4.08E-02 | 0.7618 | 2.65E-04 | 0.3948 | 3.28E-02 |
| **HSPA5** | Endoplasmic reticulum chaperone BiP | -0.0159 | 4.45E-01 | 0.5329 | 1.53E-02 | 0.5488 | 4.45E-03 |
| **TKT** | Transketolase | 1.1727 | 2.31E-05 | 1.5903 | 3.81E-07 | 0.4176 | 4.27E-01 |
| **HSP90AA1** | Heat shock protein HSP 90-alpha | 0.2849 | 2.93E-01 | 0.5383 | 1.85E-02 | 0.2534 | 5.21E-01 |
| **GOT1** | Aspartate aminotransferase | 0.8009 | 5.08E-03 | 1.2563 | 5.93E-05 | 0.4554 | 3.44E-01 |
| **GSR** | Glutathione reductase | 0.6505 | 3.69E-03 | 1.0289 | 3.53E-05 | 0.3784 | 6.54E-02 |
| **PSMA3** | Proteasome subunit alpha type | 0.9273 | 1.37E-03 | 1.0851 | 4.08E-04 | 0.1578 | 5.76E-01 |
| **PEPD** | Xaa-Pro dipeptidase | 0.9544 | 9.97E-04 | 1.3416 | 3.14E-05 | 0.3872 | 5.66E-01 |
| **FAH** | Fumarylacetoacetase | 0.9875 | 1.72E-04 | 1.1781 | 1.16E-05 | 0.1906 | 8.44E-01 |
| **PSMA7** | Proteasome subunit alpha type | 0.6136 | 1.24E-02 | 1.3233 | 1.64E-06 | 0.7097 | 9.67E-03 |
| **PSMA5** | Proteasome subunit alpha type | 0.1565 | 4.66E-01 | 0.9201 | 2.14E-04 | 0.7636 | 6.05E-03 |
| **PSMA4** | Proteasome subunit alpha type-4 | 0.1937 | 2.49E-01 | 1.5409 | 5.20E-08 | 1.3472 | 1.48E-05 |
| **HPD** | 4-hydroxyphenylpyruvate dioxygenase | 1.3441 | 2.13E-04 | 1.5215 | 4.60E-05 | 0.1774 | 1.00E+00 |
| **PSMB1** | Proteasome subunit beta type-1 | -0.1695 | 1.86E-01 | 0.9199 | 1.27E-04 | 1.0894 | 2.14E-04 |
| **PSMA6** | Proteasome subunit alpha type | 0.3648 | 3.71E-02 | 1.0520 | 1.01E-06 | 0.6871 | 2.52E-03 |
| **ACY1** | Aminoacylase-1 | 1.0812 | 3.01E-03 | 1.3908 | 2.74E-04 | 0.3096 | 8.04E-01 |
| **CAT** | Catalase | -0.0473 | 4.04E-01 | 0.8254 | 1.76E-03 | 0.8727 | 5.52E-03 |
| **PSMA1** | Proteasome subunit alpha type-1 | -0.0981 | 4.48E-01 | 0.1783 | 3.78E-01 | 0.2765 | 7.30E-01 |
| **DCXR** | L-xylulose reductase | -0.2260 | 3.86E-01 | 0.4524 | 1.79E-01 | 0.6784 | 1.13E-01 |
| **PSMA2** | Proteasome subunit alpha type | -0.5345 | 4.09E-03 | 0.7793 | 9.23E-05 | 1.3138 | 8.56E-07 |
| **FGB** | Fibrinogen beta chain | 1.9820 | 6.24E-03 | 1.3109 | 4.70E-02 | -0.6711 | 8.42E-01 |
| **SERPINA1** | Alpha-1-antitrypsin | -4.4807 | 7.69E-16 | -4.2481 | 1.93E-15 | 0.2326 | 5.44E-02 |
| **APOH** | Beta-2-glycoprotein 1 | -4.1125 | 2.66E-10 | -4.0319 | 3.50E-10 | 0.0806 | 1.00E+00 |
| **ITIH2** | Inter-alpha-trypsin inhibitor heavy chain H2 | -3.3200 | 3.21E-14 | -3.1545 | 1.42E-13 | 0.1655 | 2.58E-01 |
| **AGT** | Angiotensinogen | -3.3007 | 3.79E-11 | -3.2781 | 6.68E-11 | 0.0226 | 1.00E+00 |
| **SERPINA3** | Alpha-1-antichymotrypsin | -3.4382 | 4.28E-12 | -3.4019 | 5.32E-12 | 0.0363 | 1.00E+00 |
| **CLU** | Clusterin | -5.0626 | 2.02E-15 | -4.6916 | 7.35E-15 | 0.3710 | 5.44E-02 |
| **RBP4** | Retinol-binding protein 4 | -2.3186 | 2.13E-12 | -1.6601 | 2.87E-09 | 0.6585 | 6.35E-07 |
| **CFB** | Complement factor B | -3.7919 | 8.07E-15 | -4.0777 | 2.10E-15 | -0.2858 | 6.40E-01 |
| **VTN** | Vitronectin | -3.8019 | 7.43E-14 | -3.6456 | 1.78E-13 | 0.1563 | 1.94E-01 |
| **AZGP1** | Zinc-alpha-2-glycoprotein | -4.1348 | 2.13E-12 | -4.1014 | 2.21E-12 | 0.0334 | 7.96E-02 |
| **SERPINC1** | Antithrombin-III | -3.4197 | 3.33E-15 | -3.1922 | 1.84E-14 | 0.2275 | 5.21E-02 |
| **C4B** | Complement C4-B | -4.3479 | 6.06E-11 | -3.8301 | 8.35E-10 | 0.5178 | 1.25E-02 |
| **A1BG** | Alpha-1B-glycoprotein | -4.2279 | 1.20E-15 | -4.0527 | 2.10E-15 | 0.1752 | 2.46E-01 |
| **ORM1** | Alpha-1-acid glycoprotein | -3.1753 | 1.27E-11 | -3.4403 | 2.43E-12 | -0.2650 | 7.84E-01 |
| **CPN2** | Carboxypeptidase N subunit 2 | -4.5662 | 7.01E-10 | -4.7503 | 2.61E-10 | -0.1841 | 3.33E-01 |
| **SERPIND1** | Heparin cofactor 2 | -2.7776 | 1.00E-09 | -3.3773 | 1.90E-11 | -0.5997 | 3.28E-02 |
| **ITIH3** | Inter-alpha-trypsin inhibitor heavy chain H3 | -2.9678 | 7.87E-10 | -3.4734 | 4.92E-11 | -0.5056 | 3.33E-01 |
| **SERPING1** | Plasma protease C1 inhibitor | NA | NA | NA | NA | -0.1445 | 1.00E+00 |
| **CPN1** | Carboxypeptidase N catalytic chain | -3.2595 | 2.98E-08 | -3.5857 | 9.08E-09 | -0.3262 | 1.00E+00 |
| **LBP** | Lipopolysaccharide-binding protein | NA | NA | NA | NA | 0.1789 | 6.66E-01 |
| **KNG1** | Kininogen-1 | NA | NA | NA | NA | -0.3515 | 8.73E-01 |
| **C8G** | Complement component C8 gamma chain | NA | NA | NA | NA | -0.4907 | 3.95E-01 |
| **ITIH4** | Inter-alpha-trypsin inhibitor heavy chain H4 | NA | NA | NA | NA | -1.3199 | 3.11E-01 |

^a^Antoine, D. J., et al. 2013. *Hepatology*, 58(2), 777–787. doi: 10.1002/hep.26294

^b^Church, RJ, et al. 2019. *Hepatology*, 69(2), 760–773. doi:10.1002/hep.29802

^c^Dear, JW, et al. 2018. *The Lancet Gastroenterology & Hepatology*, 3(2), 104–113. doi:10.1016/S2468-1253(17)30266-2

^d^Peta, V, et al. 2017. *PLOS ONE*, 12(12), e0189436. doi: 10.1371/journal.pone.0189436

^e^Ravindra, KC, et al. 2023. *Nature Communications*, 14(1), 1215. doi: 10.1038/s41467-023-36858-6

^f^Remih, K, et al. 2025. *JHEP Reports*, 7(5), 101338. doi: 10.1016/j.jhepr.2025.101338

^g^Schwämmle, V, et al. 2020. *Molecular & Cellular Proteomics*, 19(8), 1396–1408. doi:10.1074/mcp.RA119.001777

**Supplementary Table 2.** Fold-change (FC) and false discovery rate (FDR) of top regulated proteins. Statistical significance was calculated using PolySTest^a^. NA, non-applicable.

| **Gene name** | **Protein name** | **APAP-injury *vs.* APAP-recovery** | | **APAP-injury *vs.* non-injured** | | **APAP-recovery *vs.* non-injured** | | **Obs.** |
| --- | --- | --- | --- | --- | --- | --- | --- | --- |
|  |  | **Log_2_(FC)** | **FDR** | **Log_2_(FC)** | **FDR** | **Log_2_(FC)** | **FDR** |  |
| **NASP** | Nuclear autoantigenic sperm protein | 2.3808 | 2.346E-06 | 3.5725 | 5.562E-09 | 1.1916 | 3.711E-02 | Upregulated in injury conditions |
| **GSS** | Glutathione synthetase | 1.5532 | 1.668E-04 | 2.6340 | 2.078E-07 | 1.0807 | 2.763E-02 |  |
| **PDXK** | Pyridoxal kinase | 1.0353 | 5.264E-04 | 2.2585 | 9.831E-08 | 1.2232 | 2.893E-03 |  |
| **HNRNPA1** | Heterogeneous nuclear ribonucleoprotein A1 | 0.6001 | 1.292E-02 | 2.1975 | 1.367E-08 | 1.5974 | 1.908E-05 |  |
| **SORD** | Sorbitol dehydrogenase | 1.2538 | 2.341E-05 | 2.1166 | 3.649E-08 | 0.8629 | 2.372E-02 |  |
| **TXNRD1** | Thioredoxin reductase 1 | 1.0608 | 5.934E-04 | 2.0642 | 9.831E-08 | 1.0035 | 1.078E-02 |  |
| **SEC13** | Protein SEC13 homolog | 0.6963 | 1.018E-03 | 1.9660 | 2.130E-10 | 1.2697 | 1.658E-05 |  |
| **GNB2L1** | Guanine nucleotide-binding protein subunit beta-2-like 1 | 0.8334 | 2.614E-02 | 1.8868 | 6.711E-06 | 1.0534 | 2.001E-02 |  |
| **SF3B3** | Splicing factor 3B subunit 3 | 0.5219 | 2.031E-02 | 1.8637 | 4.418E-08 | 1.3418 | 1.999E-04 |  |
| **CES2** | Carboxylic ester hydrolase | 0.0791 | 7.529E-01 | 1.8127 | 2.751E-04 | 1.7336 | 1.826E-03 |  |
| **HPD** | 4-hydroxyphenylpyruvate dioxygenase | 1.3441 | 2.131E-04 | 1.5215 | 4.598E-05 | 0.1774 | 1.000E+00 | Liver-enriched plasma proteins upregulated in injury conditions |
| **MAT1A** | S-adenosylmethionine synthase isoform type-1 | 0.6276 | 5.078E-03 | 0.8162 | 1.900E-04 | 0.1886 | 6.098E-01 |  |
| **CES1** | Liver carboxylesterase 1 | -0.6084 | 2.594E-02 | 0.7698 | 3.218E-03 | 1.3782 | 9.081E-05 |  |
| **FGB** | Fibrinogen beta chain | 1.9820 | 6.240E-03 | 1.3109 | 4.697E-02 | -0.6711 | 8.418E-01 |  |
| **MTHFD1** | C-1-tetrahydrofolate synthase | 0.4576 | 1.331E-01 | 0.6017 | 4.594E-02 | 0.1441 | 1.000E+00 |  |
| **ASL** | Argininosuccinate lyase | 0.8721 | 3.445E-02 | -0.0777 | 6.023E-01 | -0.9498 | 1.526E-01 |  |
| **ADH4** | Alcohol dehydrogenase 4 | 0.6098 | 1.207E-01 | 0.8864 | 1.304E-02 | 0.2766 | 7.484E-01 |  |
| **SERPINA1** | Alpha-1-antitrypsin | -4.4807 | 7.690E-16 | -4.2481 | 1.931E-15 | 0.2326 | 5.437E-02 | Upregulated in APAP-recovery (*vs.* APAP-injury) |
| **APOH** | Beta-2-glycoprotein 1 | -4.1125 | 2.660E-10 | -4.0319 | 3.505E-10 | 0.0806 | 1.000E+00 |  |
| **AMBP** | Alpha-1-microglobulin | -4.4116 | 1.989E-17 | -3.9956 | 1.395E-16 | 0.4159 | 7.961E-02 |  |
| **SERPINF1** | Pigment epithelium-derived factor | -4.0226 | 1.408E-14 | -4.3504 | 2.098E-15 | -0.3278 | 9.858E-03 |  |
| **CLU** | Clusterin | -5.0626 | 2.018E-15 | -4.6916 | 7.351E-15 | 0.3710 | 5.437E-02 |  |
| **LCN2** | Neutrophil gelatinase-associated lipocalin | -3.9812 | 2.062E-05 | -4.0451 | 1.943E-05 | -0.0639 | 1.000E+00 |  |
| **APOB** | Apolipoprotein B-100 | -6.4848 | 2.829E-15 | -5.7732 | 3.790E-14 | 0.7116 | 2.857E-04 |  |
| **AZGP1** | Zinc-alpha-2-glycoprotein | -4.1348 | 2.130E-12 | -4.1014 | 2.208E-12 | 0.0334 | 7.961E-02 |  |
| **C4B** | Complement C4-B | -4.3479 | 6.058E-11 | -3.8301 | 8.354E-10 | 0.5178 | 1.252E-02 |  |
| **GDF15** | Growth differentiation factor 15 | -4.3763 | 4.029E-09 | -3.4302 | 2.284E-07 | 0.9461 | 3.276E-02 |  |
| **CTGF** | Connective tissue growth factor | -5.7146 | 7.174E-11 | -3.3144 | 1.043E-05 | 2.4002 | 7.409E-06 |  |
| **A1BG** | Alpha-1B-glycoprotein | -4.2279 | 1.202E-15 | -4.0527 | 2.098E-15 | 0.1752 | 2.460E-01 |  |
| **MATN3** | Matrilin-3 | -3.9168 | 2.287E-08 | -4.2777 | 3.217E-09 | -0.3609 | 6.247E-01 |  |
| **CPE** | Carboxypeptidase E | -4.0735 | 2.489E-15 | -4.5716 | 3.095E-16 | -0.4980 | 2.294E-02 |  |
| **CPN2** | Carboxypeptidase N subunit 2 | -4.5662 | 7.006E-10 | -4.7503 | 2.611E-10 | -0.1841 | 3.333E-01 |  |
| **RELN** | Reelin | -4.0858 | 1.206E-07 | -4.2382 | 7.476E-08 | -0.1524 | 7.402E-01 |  |
| **SERPINA5** | Plasma serine protease inhibitor | -4.3184 | 9.814E-10 | -4.9024 | 6.746E-11 | -0.5840 | 3.276E-02 |  |
| **C1S** | Complement C1s subcomponent | -5.3533 | 1.928E-11 | -5.6642 | 4.800E-12 | -0.3109 | 4.189E-01 |  |
| **TIMP1** | Metalloproteinase inhibitor 1 | -4.7889 | 1.134E-08 | -4.0643 | 1.917E-07 | 0.7246 | 2.071E-01 |  |
| **C5** | Complement C5 | -5.5667 | 2.813E-14 | -5.8237 | 1.365E-14 | -0.2570 | 1.000E+00 |  |
| **C3** | Complement C3 | -3.4849 | 5.210E-15 | -3.5984 | 2.355E-15 | -0.1135 | 4.043E-01 |  |
| **S100P** | Protein S100-P | 3.0773 | 2.800E-05 | NA | NA | NA | NA | Unique in injury conditions |
| **ARHGDIA** | Rho GDP-dissociation inhibitor 1 | 1.0837 | 2.683E-04 | NA | NA | NA | NA |  |
| **ARF1** | ADP-ribosylation factor 1 | 2.1276 | 7.509E-08 | NA | NA | NA | NA |  |
| **PFN2** | Profilin | 1.2441 | 2.001E-06 | NA | NA | NA | NA |  |
| **THUMPD1** | THUMP domain-containing protein 1 | 2.4047 | 9.765E-08 | NA | NA | NA | NA |  |
| **AK1** | Adenylate kinase isoenzyme 1 | 1.2487 | 4.368E-05 | NA | NA | NA | NA |  |
| **TBCB** | Tubulin-folding cofactor B | 1.3532 | 9.668E-06 | NA | NA | NA | NA |  |
| **TTC38** | Tetratricopeptide repeat protein 38 | 1.1955 | 7.503E-04 | NA | NA | NA | NA |  |
| **EIF3I** | Eukaryotic translation initiation factor 3 subunit I | 1.4690 | 2.993E-05 | NA | NA | NA | NA |  |
| **EIF4E** | Eukaryotic translation initiation factor 4E | 1.0023 | 3.358E-03 | NA | NA | NA | NA |  |

^a^Schwämmle, V, et al. 2020. *Molecular & Cellular Proteomics*, 19(8), 1396–1408. doi:10.1074/mcp.RA119.001777
